# Shelter hospital mode: How do we prevent COVID-19 hospital-acquired infection?

**DOI:** 10.1017/ice.2020.97

**Published:** 2020-03-30

**Authors:** Yong Yang, Hailian Wang, Kang Chen, Jun Zhou, Shaoping Deng, Yi Wang

**Affiliations:** 1 Department of Pharmacy, Sichuan Academy of Medical Science and Sichuan Provincial People’s Hospital, Chengdu, Sichuan, China; 2 Ziyang People’s Hospital, Ziyang, Sichuan, China; 3 Hanyang Shelter Hospital, Wuhan, Hubei, China; 4 Center for Organ Transplantation, Sichuan Academy of Medical Science and Sichuan Provincial People’s Hospital, Chengdu, Sichuan, China; 5 Clinical Immunology Translational Medicine Key Laboratory of Sichuan Province, Sichuan Academy of Medical Science and Sichuan Provincial People’s Hospital, Chengdu, Sichuan, China; 6 Department of Emergency Rescue, Sichuan Academy of Medical Science and Sichuan Provincial People’s Hospital, Chengdu, Sichuan, China; 7 Emergency Rescue Department, Wenjiang Hospital, Chengdu, Sichuan, China; 8 Center for Health Management, Sichuan Academy of Medical Science and Sichuan Provincial People’s Hospital, Chengdu, Sichuan, China

*To the Editor—*With the rapid outbreak of coronavirus infection 2019 (COVID-19), as of March 15, 2020, 49,999 cases have been confirmed in Wuhan city. It has been impossible to admit all of these patients to existing hospitals in Wuhan. The best solution has been to build shelter hospitals in open areas such as stadiums or exhibition centers. However, concerns have arisen regarding hospital-acquired infections (HAIs). As of on February 5, 2020, >12,000 beds have been built, serving ~9,000 inpatients. With such a large number of patients, how can HAIs be prevented?

On February 22, 2020, at the press conference of the State Council, 3,019 healthcare personnel (HCP) were reported to have been infected. Among them, 1,716 HCP had confirmed infection and 5 HCP had died of COVID-19. In Wuhan city, there were 1,080 infected HCP. However, at the shelter hospitals, with >5,000 HCP, none had been infected between February 5 and this letter. How were HCP HAIs prevented? We report here the following measures taken at the shelter hospitals.

## 1. Disinfection of clean areas, semicontaminated, and contaminated areas

For the contaminated areas, disinfection is performed 4 times daily: the environment, air, floor and the surface of tables are sprayed with a 2,000 mg/L chlorine-containing disinfectant for no less than 30 minutes. For patient vomitus and secretions, the areas are cleaned, and then the contaminated ground is sprayed with 2,000 mg/L chlorine-containing disinfectant. Medical waste and other wastes are placed in double-layered yellow garbage bags, which are tightly closed.

For semicontaminated and clean areas, the disinfectant contains 500 mg/L chlorine, and disinfection is performed twice daily. However, if an area is contaminated with blood or vomit, the floor is cleaned then disinfected with 2,000 mg/L chlorine-containing disinfectant for 30 minutes. Air disinfection is performed using 3 methods: (1) A window is opened and the area ventilated for no less than 30 minutes 2–3 times daily. (2) Ultraviolet irradiation is applied for 30 minutes (twice daily). (3) Areas are sprayed with 500 mg/L chlorine-containing disinfectant for >30 minutes.

## 2. Patient-related decontamination

All patients are given new face masks every day. The patient living area is disinfected 4 times daily. For discharged patients, all personal items are sprayed with 75% EtOH. These patients change into clean clothes brought by their families after taking a hot bath for at least 30 minutes. All the remaining clothes are disinfected and discarded as medical waste. Subsequently, in clean areas, clothes worn by the patient are disinfected again prior to discharge. For items in the contaminated area, used sheets and bedding are disinfected and discarded. Other items, such as mattresses, are disinfected and freshly cleaned bedding and sheets are provided for newly admitted patients. Glasses, mobile phones, keys, credit cards, and other items are sprayed with 75% EtOH.

## 3. Healthcare personnel related disinfection

Before entering the shelter hospital, all HCPs don protective equipment in the following sequence: white coats, N95 facial masks, surgical masks, surgical hats, protective goggles, shoe covers, isolation gowns, gloves, protective suits, another pair of gloves, protective hoods, and boot covers. All staff entering and exiting the shelter hospital are recorded.

For exiting the shelter hospital, several steps are required. First, the HCP enter the buffer room, where they perform hand disinfection and spray 75% EtOH over all protective clothing again. After this step, they enter the first changing room, which is also considered to be contaminated. In this room, they take off the first layer of gloves and put on new clean gloves to take off the protective hood, protective suit, protective goggles, and surgical mask, sequentially. After taking off each protective item, they repeat hand hygiene. After taking off the surgical mask and disinfecting the hands, the HCP enters the second changing room, which is considered semicontaminated. In that room, they take off the isolation gown, surgical hat, N95 face mask and gloves. In addition, hands are disinfected frequently and then put on a clean surgical mask to enter the clean area. At the clean area, the body temperature is determined and recorded.

## 4. Occupational exposure

Occupational exposure includes skin and mucosa, respiratory exposure, and needle sticks from confirmed patients. For skin exposure, disinfection with 0.5% iodine or H_2_O_2_ for 3 minutes is performed then wiped off with clean water. For mucosal exposure, HCP are required to rinse the exposure site with 0.9% saline or 0.05% iodine. For needle sticks, HCP squeeze the blood out and rinse the wound with flowing water then sterilize with 75% EtOH or 0.5% iodine. For respiratory exposure, the mouth and nose of HCP are protected by a facemask within 1 m of an unmasked confirmed patient. For damaged gloves, HCP should are required to disinfect the hands with 0.5% iodine or H_2_O_2_ for 3 minutes and then rinse with copious water. Finally, HCP are required to leave the contaminated area and to report exposures to infection control personnel.

By following all of the strategies listed here, we successfully prevented HAIs at shelter hospitals, which have >900 patients per open area in each hospital. Up to now, there has been no occurrence of HCP infection by COVID-19 at shelter hospitals. Therefore, our experience has proven efficacious and successful for hospital infection management and prevention during the COVID-19 outbreak.

